# Effect of Ginseng Powder Supplementation on the Physicochemical Properties, Antioxidant Capacity, and Sensory Characteristics of Cream Soup

**DOI:** 10.3390/foods11091193

**Published:** 2022-04-20

**Authors:** Kang-Sik Kwon, Umair Shabbir, Seung-Hyeon Cha, Keum-Il Jang

**Affiliations:** 1 Department of Food Science and Biotechnology, College of Agriculture and Life Sciences, Kangwon National University, Chuncheon 24341, Korea; kgs223@kangwon.ac.kr (K.-S.K.); umair336@gmail.com (U.S.); 2 Department of Food Science and Biotechnology, Chungbuk National University, Cheongju 28644, Korea; fodss3@naver.com

**Keywords:** ginseng powder, cream soup, antioxidant effect, quality characteristics, biofunctional properties

## Abstract

Ginseng has been used as a medicinal herb in Asian countries for hundreds of years. It contains many kinds of ginsenosides as major active ingredients and is known to have neuroprotective, anti-inflammatory, antitumor, and antidiabetic properties. In this study, we have developed cream soup with different concentrations (0%, 3%, 5%, 7%, and 10%) of ginseng powder (GP) and determined the quality characteristics (color, viscosity, salinity, etc.) and antioxidant activity, along with sensory parameters. After the addition of GP, significant differences in salinity, L* and a*color value, DPPH, and ABTS were found among different concentrations of GP. Cream soup supplemented with GP 10% exhibited the highest values for DPPH and ABTS (83.5% and 87%, respectively), while the contents of total phenolic and saponin were 0.651 ± 0.02 (mg Gallic acid Equiv./g, DW) and 0.797 ± 0.05 (mg Diosgenin Equiv./g, DW), respectively. Moreover, there were no significant changes for °Brix value, pH, acidity, and total flavonoids content compared to control. The sensory characteristics indicated bitterness with the increase in the concentration of GP. However, a non-significant difference was observed between the control and supplemented samples for color, viscosity, and overall preference. Therefore, the supplementation of GP to cream soup could exhibit health benefits and increase the demand for ginseng to promote public health as functional food material.

## 1. Introduction

Ginseng (*Panax ginseng* C.A. Meyer) is known as the king of herbs due to its therapeutic effects and has been used to treat diseases for years in several Asian countries. It has also gained global popularity over the last three decades and is used in medicines, cosmetics, agricultural products, and dietary supplements [1]. Ginseng has neuroprotective activity (Alzheimer’s diseases, Parkinson’s diseases, and Huntington’s disease), anti-inflammatory, antioxidative, antitumor, and cardioprotective potential [1,2,3]. The primary bioactive compounds of ginseng are ginsenosides (triterpene saponins); however, ginseng’s therapeutic effects not only depend on ginsenosides as it also contains several other bioactive compounds such as polyphenols (ferulic, cinnamic, syringic acid, etc.), volatile oils, and polyacetylene [4,5,6]. South Korea, China, the United States, and Canada are the major producers of ginseng, and its global market is expected to reach USD 11.7 billion by 2026 [7].

The supplementation of food products with natural ingredients, including ginseng, can increase the bioactivity for human health [8]. Ginseng extract was supplemented into quark cheese by Kim et al. [9], and they observed a significant increase in the antioxidant activity of quark cheese. Moreover, Shin et al. [10] examined the effect of ginseng supplementation into *cheonggukjang* (traditional Korean food) on plasma lipid profile and blood glucose level in subjects (men:women; mean age, 44.9 ± 3.1 years) with impaired fasting glucose. They found a significant decrease in the concentration of plasma non-high-density lipoprotein-cholesterol and fasting blood glucose in subjects. In another study, Abdel-Aziem and colleagues [11] supplemented whey protein with ginseng extract to rats fed an aflatoxin-contaminated diet (which contributes to several cancers and liver diseases). They observed a significant decrease in lipid peroxidation, oxidative stress, and an increase in glutathione levels in the liver and testis. Therefore, the supplementation of ginseng into food products can increase the therapeutic potential and decrease the diseases related to the toxicity of cellular mechanisms. In this study, we have supplemented ginseng powder (GP) into cream soup (as it is one of the basic and traditional dishes of the world). To our knowledge, there is still no research on the effects of GP supplemented cream soup as a new cream soup product. In the present study, we examine the physicochemical and sensory properties, changes in color and viscosity, total phenolic, flavonoid, saponin content with antioxidant effects of the cream soup supplemented with GP. Furthermore, the overall objective of this study was to add GP in the cream soup that would exhibit higher quality and antioxidant properties and may lead to the idea of a new product with healthier options for the food industry to grow the convenience of the food market.

## 2. Materials and Methods

White ginseng used in this study was four-year-old ginseng harvested from Eumseng-gun, Chungcheongbuk-do, South Korea. Roux powder used in soup production was purchased from Unilever. Co., Ltd., (Auckland, New Zealand) and onion powder, garlic powder, milk, beef powder, xanthan gum, salt, and cheese powder from Pocheon city Gyeonggi-do, South Korea.

### 2.1. Reagents

All reagents and chemicals used in this study were of analytical grade. As major extraction reagents, n-butanol, ethanol, methanol, and ethyl acetate were obtained from Merck Co. (Darmstadt, Germany). 1,1-Diphenyl-2-picrylhydrazyl (DPPH), (2,2′-azino-(3-ethybenzo thiazoline-6-sulphonic acid) (ABTS), ascorbic acid, gallic acid, quercetin, diosgenin, Na_2_CO_3_, Al(NO_3_)_3_, catechin hydrate, Folin–Ciocalteu reagents, all were purchased from Sigma-Aldrich Co. (St. Louis, MO, USA).

### 2.2. Preparation of the Sample

White ginseng was crushed into 30–50 mesh, ground for 30 s using a Shinil mixer (SMX 700 SKB), strained using the sieve (Chung Gue Co. Seoul, Korea) at 150 μm and stored in polythene bags. Moreover, we used the basic recipe to prepare cream soup with GP, and the composition ratio of cream soup was the same as produced by Ottogi Food Co., Ltd., Anyang-si, Gyeonggi-do, South Korea. GP was added with different concentrations (control: 0%, GS3: 3%, GS5: 5%, GS7: 7%, and GS10: 10%). After weighing and mixing all ingredients, 300 mL of 100 °C boiling water was added to the soup, stirred for 5 min, and kept at 25 °C before further analysis (Supplementary Table S1). Cream soups (control and supplemented with GP) were prepared in 3 different batches (using the same conditions and ingredients) to obtain the triplicate values.

### 2.3. Proximate Composition Analysis

Proximate content (crude protein, fat, ash, moisture, and titratable acidity) analysis of GP, cream soup, and cream soup added with different concentrations of GP were carried out by following the AOAC methods [12].

### 2.4. Quality Characteristics Analysis

Color values were evaluated using a colorimeter (Hunterlab, Ultrascan Pro, Reston, VA, USA). The pH value was measured using a pH meter (Thermo Electron Corp., Waltham, MA, USA). Total soluble solids were calculated using glucose meter (PR-201α, Atago Co., Tokyo, Japan) and salinity with salinometer (PAR-SALT, Atago Co., Tokyo, Japan). Moreover, viscosity was measured using the viscometer (Brookfield digital viscometer, Middleboro, MA, USA) and dietary fiber using the non-digestible residues by the digestive enzyme method.

### 2.5. Total Phenolic Content (TPC)

The TPC of the cream soup added with GP extract was analyzed using Shabbir et al. [13] with slight modifications. Briefly, 25 mL of 99% ethanol was added to 10 g of the sample, sonicated at 40 °C for 2 h, and centrifuged at 3000 rpm for 10 min to prepare a supernatant as a soup extract. Afterward, 2 mL of 2% Na_2_CO_3_ solution was mixed with the soup extract (0.1 mL) and stayed for 3 min at room temperature, then 0.1 mL of Folin–Ciocalteu (50%) was added and vortexed. After incubating at room temperature for 30 min, the absorbance was measured using a spectrophotometer (Beckman Instruments Inc., Fullerton, CA, USA) at 750 nm. The standard curve was calculated using gallic acid (Sigma Chemical Co, St. Louis, MO, USA), and the values were expressed as mg gallic acid Equiv./gram, DW.

### 2.6. Total Flavonoid Content (TFC)

The extracted solution was used for TFC analysis (extraction protocol explained in the TPC method), and the assay was conducted using the method of Tyagi et al. [14] with slight modifications. In summary, 1 mL of distilled water and 0.075 mL of 5% NaNO_2_ were added to 0.25 mL of the sample extract and incubated for 5 min. Then, AlCl_3_ (75 μL; 100 g/L) was mixed and settled for 6 min. Afterward, 600 μL of distilled water followed by 500 μL of 1 M NaOH was mixed. The absorbance was measured at 510 nm using a spectrophotometer (UV-1650PC, Shimadzu, Kyoto, Japan). The concentration of TFC was examined from the standard (catechin) and presented as mg catechin Equiv./gram, DW.

### 2.7. Total Saponin Content (TSC)

The TSC content was calculated by following the method of Kurkin et al. [15] after implying the extraction procedure (mentioned in the TPC method). The total saponin content was calculated from the Diosgenin standard curve and presented in mg Diosgenin Equiv./gram, DW.

### 2.8. Antioxidant Capacity

Antioxidant activity (DPPH and ABTS) of samples (1 mg/mL) was measured using the methods of Shabbir et al. [13] with slight changes. For the DPPH assay, 0.8 mL of DPPH radical solution was mixed with 0.2 mL of sample and incubated for 30 min in the dark at room temperature, and absorbance was checked at 517 nm. For the ABTS assay, 1 mL of ABTS radical solution was mixed with 0.05 mL of samples, and absorbance was measured at 735 nm. The results were presented using the standard curve of Trolox (0.03–1 mg/mL).

### 2.9. Sensory Analysis

Consumer sensory analysis was performed by 20 untrained panelists (12 females, 8 males, age 25–31 years) based on the ethics and safety, research, and research design approved by Institutional Review Board (protocol number CBNU-201808-BM-687-01, Korea). Quantitative descriptive analysis was performed to evaluate the differences in the sensory characteristics among cream soup samples supplemented with GP [5,16,17]. A continuous scale (7-point) was used to measure the following characteristics: color, flavor, taste, viscosity, and overall preference (1 = extremely dislike, 2 = very dislike, 3 = slightly dislike, 4 = neither like nor dislike, 5 = slightly like, 6 = very like, 7 = extremely like).Plain bread and water were provided between samples as a cleanser, and cream soup without the GP was served as the reference standard.

### 2.10. Statistical Analysis

All results were expressed as mean ± standard deviation (SD) of triplicate analysis, and the obtained data were statistically analyzed using the Graphpad Prism 8.0 (GraphPad Software, San Diego, CA, USA). One-way analysis of variance (ANOVA) and Tukey’s test at *p* < 0.05 significance level was used to find the difference in the mean values of the samples.

## 3. Results and Discussion

### 3.1. Proximate Composition and Quality Characteristics

The composition and quality characteristics of cream soup supplemented with different concentrations of GP (3%, 5%, 7%, and 10%) and cream soup without GP (control) are illustrated in Table 1. Moreover, proximate composition and quality characteristics of GP are expressed in Supplementary Table S2. The composition of cream soup without GP (control) was 74.17% moisture, 3.63% crude protein, 7.57% crude fat, 1.32% crude ash, 12.87% carbohydrates, and 5.03% total dietary fiber. Among these contents, carbohydrates were affected significantly and exhibited a dose-dependent increasing pattern by the addition of GP. However, the moisture and crude ash content did not show significant differences for GS3 (3% GP), GS5 (5% GP), and GS7 (7% GP), but GS10 (10% GP) exhibited significant difference. Moreover, significant differences in GS7 and GS10 were observed for total dietary fiber content with other treatments. In case of crude protein content, GS7 (3.75%) and GS10 (3.81%) exhibited significant differences. The results for soluble solids (°Brix), total acidity (CA eq %), and pH were not affected significantly by the addition of the GP. Furthermore, the salinity and viscosity of the control samples (5 g each) were 7.7% and 7337 cP, respectively. A dose-dependent increment was observed for salinity (GS3: 8%, GS5: 8.3%, GS7: 8.5%, GS10: 9.1%) and viscosity (GS3: 8867 cP, GS5: 8913 cP, GS7: 9380 cP, GS10: 9650 cP) for GP supplemented cream soup. The results for viscosity are consistent with Shin et al. [18], who also observed an increase in the consistency of Tarakjuk (traditional Korean soup) with the addition of ginseng. Moreover, an increase in the salinity with the amount of GP might be due to the presence of sodium content (0.62 mg/kg) in the ginseng [19].

In terms of the color parameters of the GP at different concentrations (3%, 5%, 7%, and 10%) in cream samples, L* values (lightness) significantly decreased with the increase in the concentration of GP and b* values (yellowness) were reduced with the addition of GP. However, all values were non-significant to each other with different concentrations. In contrast, a*values (redness) increased considerably (*p* < 0.05). We have used white ginseng, which might have affected the color of the cream soup. These results are in accordance with Kang et al. [20], who analyzed the quality characteristics of cookies prepared with the addition of GP.

### 3.2. TSC, TPC, and TFC of Samples

We have analyzed the content of saponin, phenolic, and flavonoids of the samples (represented in Table 2). The TPC, TFC, and TSC were measured in terms of gallic acid, catechin, and diosgenin equivalent, respectively. In the case of TSC and TPC, an increasing trend was observed with the increase in the concentration of GP into cream soup. However, GS5 and GS7 exhibited a non-significant difference for TPC. The lowest TFC (mg GAE./g) was recorded for control (0.408 ± 0.03), and highest for GS10 (0.651 ± 0.02). Moreover, the TSC (mg DE./g) in control, GS3, GS5, GS7, and GS10 was 0.405 ± 0.01, 0.465 ± 0.02, 0.546 ± 0.02, 0.685 ± 0.05, and 0.797 ± 0.05, respectively. An increase in both TSC and TPC in samples with the increase in the concentration of GP was due to the presence of these contents (in higher quantities) in GP (Supplementary Table S2). In contrast, no significant change was observed in TFC among all the samples, and this might be due to the very less presence of TFC in GP (Table S2). Current findings agreed with Kim et al. [21].

### 3.3. Antioxidant Capacity

**Figure 1:**
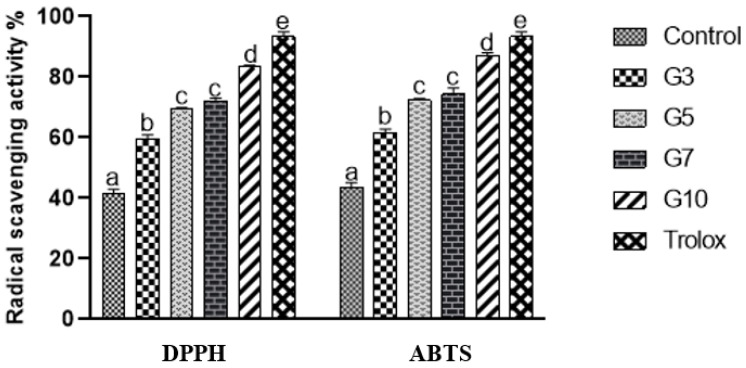
Antioxidant capacity of cream soup supplemented with ginseng powder. Two radical scavenging assays DPPH and ABTS were performed. The graphs are expressed as mean value ± S.D. of triplicate experiments in percentage. Values represent triplicate readings and different small letters in the figure denote significant differences (*p* < 0.05). Control: soup without ginseng powder; GS3: Soup with 3% ginseng powder; GS5: Soup with 5% ginseng powder; GS7: Soup with 7% ginseng powder; GS10: Soup with 10% ginseng powder.

An increase in the levels of reactive oxygen species can damage the structure of DNA, lipids, and protein and can lead to aging and related diseases [22]. Therefore, foods with higher antioxidant capacity are required to reduce/inhibit the levels of reactive species. The supplementation of foods with herbs such as ginseng can serve as a good source of antioxidants and increase the production demand of particular food. In the present study, we have analyzed the antioxidant potential of cream soup supplemented with GP and the DPPH and ABTS assays; both are known as the standard methods for measuring the antioxidant capacity of samples [13]. Figure 1 illustrates the antioxidant activity of cream soup supplemented with GP (1 mg/mL). Both of the assays showed a significant increase in the antioxidant capacity of cream soup with the increase in the concentration of GP. Minimum DPPH and ABTS radical scavenging activity were exhibited by control (42% and 44.5%, respectively) and highest by GS10 (83.5% and 87%, respectively). Similar results were noticed by Kim et al. [21] and Kim et al. [9]. The increase in the activity might be due to the higher polyphenols (saponin and various other ginsenosides) in the samples, as studies suggest they play an important role in enhancing antioxidant activity [23].

### 3.4. Sensory Evaluation

The sensory characteristics of cream soup added with different concentrations (3%, 5%, 7%, and 10%) of GP are expressed in Table 3. The acceptability of taste was negatively affected by the increase in GP, and flavor was also affected negatively for GS10. However, scores for color, viscosity, and overall preference were not affected significantly among all samples. Similar results were observed by Lee et al. [5] and Kim et al. [9]. Studies have documented that the supplementation of ginseng extract/powder into food products can increase the biofunctional activities for human health but contribute negatively to sensory scores (especially bitterness) [24]. Ginseng contains propylene glycol or triterpenoid peptides that are responsible for the bitterness of ginseng [25]. In this study, we also have found a decrease in taste scores. However, the scores for color, viscosity, flavor, and overall preference were non-significantly different, which might be due to the wide utilization of ginseng in Korean cuisines, cosmetics, and medicines.

## 4. Conclusions

Ginseng is widely acceptable for its biological activities. In this study, we developed the cream soup supplemented with different concentrations of GP and evaluated the physicochemical, quality (color, viscosity, salinity, etc.), and sensory characteristics (color, flavor, taste, viscosity, and overall preferences). Moreover, antioxidant capacity, total phenolic, flavonoid, and saponin contents were also examined. Significant increase in the antioxidant capacity and phenolics were observed after the supplementation of GP in cream soup. Therefore, we have found that different concentrations of GP can be applied to develop cream soup with higher functional activities and can be helpful to cope with several diseases caused by oxidative stress. Moreover, this study may broaden the application of ginseng in the food industry.

## Figures and Tables

**Table 1:** Physicochemical, quality, and color analysis of cream soup supplemented with ginseng powder.

Variables		Control	GS3	GS5	GS7	GS10
Moisture (%)		74.17 ± 0.06 ^a^	74.27 ± 0.06 ^a^	74.33 ± 0.12 ^a^	74.57 ± 0.15 ^ab^	74.77 ± 0.15 ^b^
Crude protein contents (%)		3.63 ± 0.73 ^a^	3.66 ± 0.33 ^a^	3.66 ± 0.05 ^a^	3.75 ± 0.05 ^b^	3.81 ± 0.02 ^c^
Crude fat contents (%)		7.57 ± 0.26 ^a^	7.58 ± 0.19 ^a^	7.59 ± 0.52 ^a^	7.60 ± 0.20 ^a^	7.61 ± 0.06 ^a^
Crude ash contents (%)		1.32 ± 0.03 ^a^	1.34 ± 0.02 ^a^	1.35 ± 0.02 ^a^	1.36 ± 0.02 ^a^	1.40 ± 0.02 ^b^
Carbohydrate (%)		12.87 ± 0.74 ^a^	13.18 ± 0.54 ^ab^	13.45 ± 0.58 ^b^	13.78 ± 0.27 ^bc^	13.92 ± 0.21 ^c^
Total dietary fiber contents (g/100 g)		5.03 ± 0.375 ^a^	5.23 ± 0.813 ^a^	5.25 ± 0.560 ^a^	5.81 ± 0.532 ^b^	5.87 ± 0.532 ^b^
Soluble solids (°Brix)		12.0 ± 0.4 ^a^	12.0 ± 0.7 ^a^	11.9 ± 0.2 ^a^	11.9 ± 0.2 ^a^	12.1 ± 0.2 ^a^
pH		6.62 ± 0.05 ^a^	6.61 ± 0.06 ^a^	6.60 ± 0.07 ^a^	6.59 ± 0.08 ^a^	6.59 ± 0.07 ^a^
Total acidity (CA eq %)		0.054 ± 0.008 ^a^	0.057 ± 0.005 ^a^	0.062 ± 0.008 ^a^	0.062 ± 0.008 ^a^	0.064 ± 0.004 ^a^
Salinity (%)		7.7 ± 0.6 ^d^	8.0 ± 0.4 ^c^	8.3 ± 0.2 ^bc^	8.5 ± 0.5 ^b^	9.1 ± 0.2 ^a^
Viscosity (cP)		7337 ± 6 ^e^	8867 ± 32 ^d^	8913 ± 15 ^c^	9380 ± 10 ^b^	9650 ± 20 ^a^
Color	L* value	93.56 ± 5.94 ^a^	83.60 ± 4.37 ^b^	79.18 ± 3.76 ^c^	80.22 ± 2.37 ^d^	78.66 ± 1.26 ^e^
	a* value	−0.81 ± 0.19 ^a^	−0.66 ± 0.05 ^b^	−0.49 ± 0.12 ^c^	−0.37 ± 0.26 ^d^	−0.40 ± 0.24 ^e^
	b* value	5.84 ± 1.44 ^a^	3.48 ± 1.21 ^b^	3.03 ± 0.61 ^b^	3.04 ± 0.77 ^b^	3.94 ± 0.91 ^b^

Means of triplicate values represented by different superscripts in the same row are significantly different at *p* < 0.05. Control: soup without ginseng powder; GS3: Soup with 3% ginseng powder; GS5: Soup with 5% ginseng powder; GS7: Soup with 7% ginseng powder; GS10: Soup with 10% ginseng powder; means of triplicate values represented by different superscripts in the same row are significantly different at *p* < 0.05. L* value (lightness), ranges from black to white, a* value (redness), ranges from green (negative) to red (positive), b* value (yellowness), ranges from blue (negative) to yellow (positive).

**Table 2:** Total phenolic content (TPC), total flavonoid content (TFC), and total saponin content (TSC) of the samples.

Samples	TPC (mg GAE/g, DW)	TFC (mg CEg, DW)	TSC (mg DE./g, DW)
Control	0.408 ± 0.03 ^a^	0.009 ± 0.03 ^a^	0.405 ± 0.01 ^a^
GS3	0.501 ± 0.01 ^b^	0.010 ± 0.01 ^a^	0.485 ± 0.02 ^b^
GS5	0.529 ± 0.02 ^ab^	0.011 ± 0.02 ^a^	0.546 ± 0.02 ^c^
GS7	0.533 ± 0.02 ^ab^	0.009 ± 0.02 ^a^	0.685 ± 0.05 ^d^
GS10	0.651 ± 0.02 ^c^	0.010 ± 0.02 ^a^	0.797 ± 0.05 ^e^

Control: soup without ginseng powder; GS3: Soup with 3% ginseng powder; GS5: Soup with 5% ginseng powder; GS7: Soup with 7% ginseng powder; GS10: Soup with 10% ginseng powder; means of triplicate values represented by different superscripts in the same column are significantly different at *p* < 0.05.

**Table 3:** Sensory evaluations of cream soup added with ginseng powder.

Variables	Control	GS3	GS5	GS7	GS10
Color	5.25 ± 0.91 ^a^	5.20 ± 0.95 ^a^	4.89±1.28^a^	5.05 ± 1.00 ^a^	5.10 ± 1.25 ^a^
Flavor	4.90 ± 0.97 ^a^	4.65 ± 1.33 ^a^	4.30±1.42^a^	4.60 ± 1.19 ^ab^	4.00 ± 0.97 ^b^
Taste	5.29 ± 1.25 ^a^	4.80 ± 1.40 ^ab^	3.90±1.53^b^	3.60 ± 1.43 ^bc^	3.35 ± 1.41 ^c^
Viscosity	4.90 ± 1.10 ^a^	4.95 ± 1.23 ^a^	5.05±1.59^a^	5.15 ± 1.10 ^a^	5.35 ± 1.45 ^a^
Overall preference	5.19 ± 1.04 ^a^	4.97 ± 1.27 ^a^	4.67±1.15^a^	4.49 ± 1.41 ^a^	4.37 ± 1.42 ^a^

Control: soup without ginseng powder; GS3: Soup with 3% ginseng powder; GS5: Soup with 5% ginseng powder; GS7: Soup with 7% ginseng powder; GS10: Soup with 10% ginseng powder; means of triplicate values represented by different superscripts in the same column are significantly different at *p* < 0.05.

## Data Availability

The date presented in this study are available in article.

## Supplementary Materials

The following supporting information can be downloaded at: https://www.mdpi.com/article/10.3390/foods11091193/s1, Table S1. The formula for cream soup preparation added with ginseng powder. Table S2. Proximate composition, quality, and antioxidant properties of ginseng powder.

## Abbreviations

GP	Ginseng powder
TPC	Total phenolic content
TFC	Total flavonoid content
TSC	Total saponin content
SD	Standard deviation
ANOVA	Analysis of variance
