# Supplementary material for: Effect of Ginseng Powder Supplementation on the Physicochemical Properties, Antioxidant Capacity, and Sensory Characteristics of Cream Soup
Source: Foods. 2022 Apr 20;11(9):1193. doi: 10.3390/foods11091193 (PMC9105560; doi:10.3390/foods11091193)
Supplement: Supplementary file 1 [file foods-11-01193-s001.zip › foods-1667424-supplementary.pdf]

Table S1. The formula for cream soup preparation added with ginseng powder

(unit : g)

Ingredients	Control	GS3	GS5	GS7	GS10
Onion powder	2.7	2.7	2.7	2.7	2.7
Garlic powder	1.8	1.8	1.8	1.8	1.8
Cheeze powder	5	5	5	5	5
Roux powder[1]	55	52	50	48	45
Beef powder	3	3	3	3	3
Milk powder	30	30	30	30	30
Salt	1	1	1	1	1
White pepper	1	1	1	1	1
Xanthan gum	0.5	0.5	0.5	0.5	0.5
Ginseng powder	0	3	5	7	10
Total	100	100	100	100	100
Water(mL)	300	300	300	300	300

Control: soup without ginseng powder, GS3: Soup with 3% ginseng powder, GS5: Soup with 5% ginseng powder, GS7: Soup with 7% ginseng powder, GS10: Soup with 10% ginseng powder.

Table S2. Proximate composition, quality, and antioxidant properties of ginseng powder

Variables		Ginseng powder
Moisture contents (%)		8.66±0.07
Crude protein contents (%)		12.25±0.04
Crude fat contents (%)		2.23±0.05
Crude ash contents (%)		3.66±0.01
Carbohydrate contents (%)		73.19±0.03
DPPH Radical scavenging ability (%)		40.82±1.43
ABTS Radical scavenging ability (%)		48.34±2.72
Total polyphenol contents (mg Gallic acid Equiv./ g, DW)		0.312±0.036
Total flavonoid contents (mg Catechin Equiv./g, DW)		0.071±0.000
pH		5.46±0.01
Total acidity (CA eq %)		3.737±0.185
Total saponin (mg Diosgenin Equiv./g, DW)		43.045±5.141
Chromaticity	L* value	86.37±0.30
	a* value	-0.08±0.01
	b* value	-14.66±0.13

All data are mean±SD of triplicate analyses. L* value (lightness), ranges from black to white, a* value (redness), ranges from green (negative) to red (positive), b* value (yellowness), ranges from blue (negative) to yellow (positive).
